# Toward a New Generation of Photonic Humidity Sensors

**DOI:** 10.3390/s140303986

**Published:** 2014-02-26

**Authors:** Stanislav A. Kolpakov, Neil T. Gordon, Chengbo Mou, Kaiming Zhou

**Affiliations:** School of Engineering and Applied Science, Aston University, Aston Triangle, Birmingham B4 7ET, UK; E-Mails: n.gordon@aston.ac.uk (N.T.G.); c.mou1@aston.ac.uk (C.M.); k.zhou@aston.ac.uk (K.Z.)

**Keywords:** humidity sensor, relative humidity, adsorption dynamic analysis, acoustic, electronic, optic

## Abstract

This review offers new perspectives on the subject and highlights an area in need of further research. It includes an analysis of current scientific literature mainly covering the last decade and examines the trends in the development of electronic, acoustic and optical-fiber humidity sensors over this period. The major findings indicate that a new generation of sensor technology based on optical fibers is emerging. The current trends suggest that electronic humidity sensors could soon be replaced by sensors that are based on photonic structures. Recent scientific advances are expected to allow dedicated systems to avoid the relatively high price of interrogation modules that is currently a major disadvantage of fiber-based sensors.

## 1. Introduction

Water is the most essential chemical compound for humans on the Earth. Life, as we know it, is impossible without water. More than 70% of the surface of our planet is covered by water. Water is present everywhere, in air, in soil, in rocks, in plants and an animals. In air or in other gasses water can exist in two different forms. The term moisture refers to water in liquid form that is suspended in air or gas in form of small droplets. The term humidity refers to the concentration of water vapor in air, where the water is in a gaseous phase.

**Figure 1.**
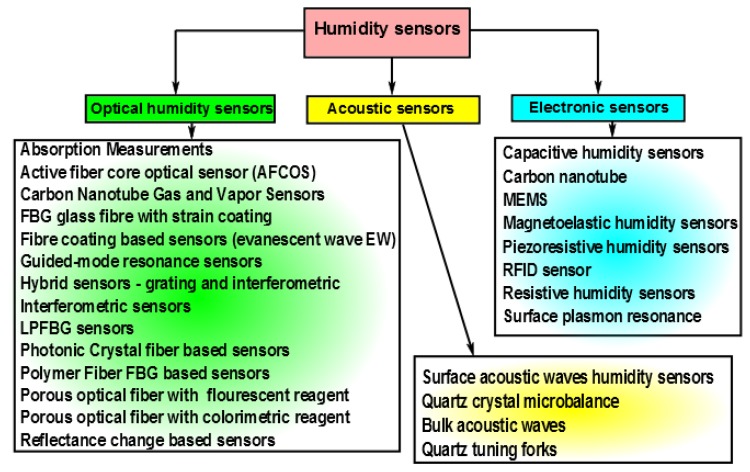
Types of humidity sensors.

Humidity is a physical quantity that has significant importance in a number of areas ranging from life sciences [1,2] to building automation [3]. Hence humidity control, sensing and monitoring is important in a number of areas. Fast humidity sensors are required for the diagnosis of pulmonary diseases [4] and for mapping the human respiratory system [5] by monitoring the water vapor content of exhaled breath. For meteorological applications [6] sensing of humidity is important as it indicates the likelihood of precipitation, dew or fog. In the semiconductor industry, the performance of photo-resist is critically dependent on the humidity. In the electronics industry, humidity monitoring is important as electronic items may malfunction due to high humidity. Furthermore, humidity control is essential in some buildings where humidity sensitive materials are stored such as museums, archives, warehouses. For human comfort and to maintain the quality of a number of food products, it is important to control humidity levels inside buildings, cars, shops and other places. Many different types of humidity sensors are needed to cover all the previously mentioned applications. As a consequence, a wide range of sensor types (see Figure 1) has been proposed for humidity measurements.

In Figure 1, humidity sensors have been organized into three groups. Electronic sensors are the most common type of sensors today. This technology has a very long history and follows the first generation of mechanical humidity sensors. These mechanical sensors were based mainly on change in the mechanical properties of some materials. These materials were frequently of animal origin, for example, horse or human hairs. These first mechanical sensors, which were slow and imprecise, were used throughout human history until the second half of twentieth century. At that time, practically simultaneously with the first electronic chips, the second generation of the humidity sensors has emerged. These were electronic humidity sensors. Today, humidity sensing based on electronic sensors is the dominant technology.

More recently, research has been carried out on the use of acoustic waves to measure humidity. So far, this research has not led to the development of new sensors and little research is currently being reported in this area in comparison with other humidity sensing areas. However, a third generation of sensors has now emerged with the development of fiber technologies. These sensors, which are mainly based on interferometric techniques, are faster and more robust than the electronic ones. This technology, which has been developed over the past twenty years, is now making its first attempts to compete with the well-established electronic one. Today, humidity sensors based on fiber interferometers still have a relatively high cost in comparison with electronic ones. However the fiber sensors have some important advantages. These sensors do not generate electrical sparks because the optical humidity sensors do not use electricity in the sensor head. This allows the use of optical humidity sensors in chemical industry, where flammable solvents are frequently employed.

**Figure 2.**
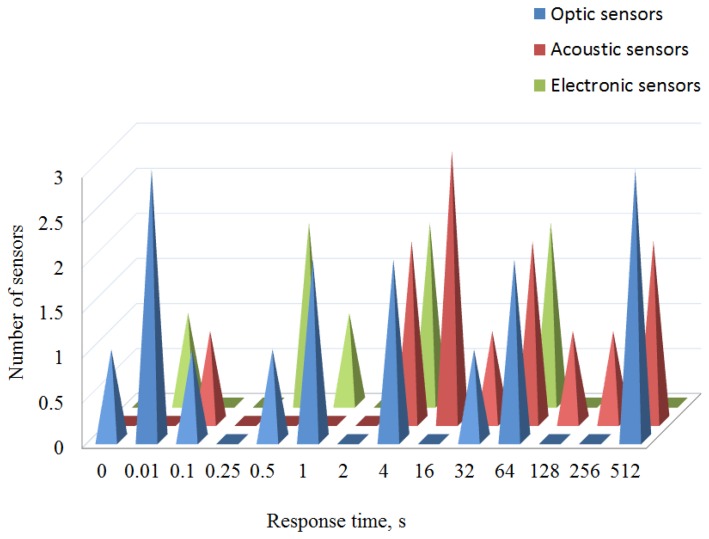
Response times of different types of humidity sensors.

A comparison between response time of different types of humidity sensors is illustrated in Figure 2. The acoustic sensors (red color) are slowest group. The second group (green color) are experimental and commercially available electronic sensors. Finally, the blue color illustrates the performance of optical humidity sensors. The fastest sensors, which are depicted on the left side of Figure 2, are interferometric sensors and have a response time of less than one second. These sensors are based on photonic crystal fibers and use poly-vinyl-alcohol as the hydrophilic material.

Current research draws attention to the fact that important technological advances have been made during the last ten years in all competing branches of humidity sensing technologies. A new generation of nano-technology based humidity sensors, complimentary metal oxide semiconductor micro-electro-mechanical system (CMOS-MEMS) humidity sensors have been investigated and demonstrated [7]. These sensors have an extremely fast response time in comparison with the electronic sensors that prevail in the market. The main advantages of these devices are their simplicity and the availability of low cost interrogation modules. However, the sensitive head of these sensors is expensive to produce in small quantities. Another kind of optical humidity sensor, which is based on nano-capillary interferometer [8–11] has been widely investigated in scientific literature. This sensor has a very simple and cheap sensing element. The manufacturing of these elements does not require a large investment in machinery, allowing on demand production. One purpose of this article is to examine the most current research in the area of humidity sensing. This review offers new perspectives and highlights an area in need of further research. Moreover, the report includes a review of recent scientific literature mainly covering the last ten years. The second section of the article is devoted to a review of the latest advances in the field of electronic humidity sensing. Progress in the field of acoustic humidity sensing is examined in the next section. Finally, in the forth section, the state-of-art in optical humidity sensing is described. The major findings indicate that a new generation of humidity sensing technology based on optical fibers is emerging.

## 2. Electronic Humidity Sensors

This section discusses the main characteristics of state-of-the-art electronic sensors for humidity detection and recent research in this area. Today these sensors are the dominant technology on the world-wide market. They detect humidity by measuring changes in the electrical characteristics of a humidity-sensitive thin film. The relatively simple design and low price of the interrogation module are the two main advantages of electronic sensors. On the other side of the coin their disadvantages are: the need for regular calibration; the difficulty in measuring relative humidity below 5% level; poor linearity and relatively long response time, which typically is of several tenth of seconds or even minutes. Moreover, the use of electronic humidity sensors in certain critical environments, remote places, potentially explosive atmospheres and areas with high electromagnetic interference is either difficult or some times impossible.

Research over the past ten years has been largely aimed at improving these characteristics and this will be discussed in the remainder of this section. In the electronic sensors, water vapor is absorbed into some hydrophilic layer and this changes the impedance of the device. Contacts are applied to the layer to measure this change. Commercially available humidity sensors are briefly reviewed in the first subsection. In the following subsections, the detectors will be classified by whether changes are measured in the capacitance or the resistance of the sensor. The second subsection is dedicated to a description of the experimental advances in the field of capacitative humidity sensors. Finally, humidity sensors that are based on resistivity changes are illustrated in the last subsection.

### 2.1. Commercially Available Humidity Sensors

Electronic humidity sensors, which are typically available for less than $10, are the most commonly available low cost sensors today. Despite the low cost, these devices are quite sophisticated. Most of these sensors can be connected directly to a microprocessor without requiring an external amplifier or digitizer. The integration of a temperature sensor allows the measured data to be corrected for temperature variations. In addition, an external microprocessor can calculate both the dew point and the concentration of water vapor. However, these commercial devices fall short of requirements in some areas as is listed below:

- too long response time in some applications
- a poor accuracy, which is close to ±2%, especially at low and high humidity
- wide hysteresis and poor linearity
- insufficient temperature operational range and bad long term stability.

The response time of these sensors is typically in the range 5–60 s. Although this is adequate for many applications, it is too long in some other areas such as for a breathing sensor. The accuracy is limited to a few percent RH and is worse in the extreme ranges of 0%–10% RH and 90%–100% RH. The devices have hysteresis, in other words the detector output for a given humidity depends on whether the humidity is increasing or decreasing. The maximum width of this hysteresis is typically a few percent RH. The maximum operating temperature is in the range 80–120 °C which is not high enough for some industrial drying applications. Finally, if the temperature of a humidity sensor drops below the dew point, condensation will prevent it operating until the temperature of the sensor has been increased for long enough for the water to evaporate. This is a common problem with humidity sensors. Some examples of performance of commercially available sensors are summarized in Table 1.

### 2.2. Capacitive Humidity Sensors

Capacitive sensors are typically produced by depositing a thin layer of a sensitive material on to closely spaced electrodes. Sometimes, these electrodes take the form of two interdigitated comb structures to increase the capacitance. This allows water vapor to interact with the top surface of the sensitive layer. Moreover, the interdigitated electrodes are simpler to produce using photolithography than a structure with electrodes on both sides. Capacitive sensors have the advantages of low power requirements and a high output signal. Clearly, the performance of a capacitive sensor will depend critically on the sensitive layer used. Table 2 lists the properties of capacitance sensors using different sensitive layers.

**Figure 3.**
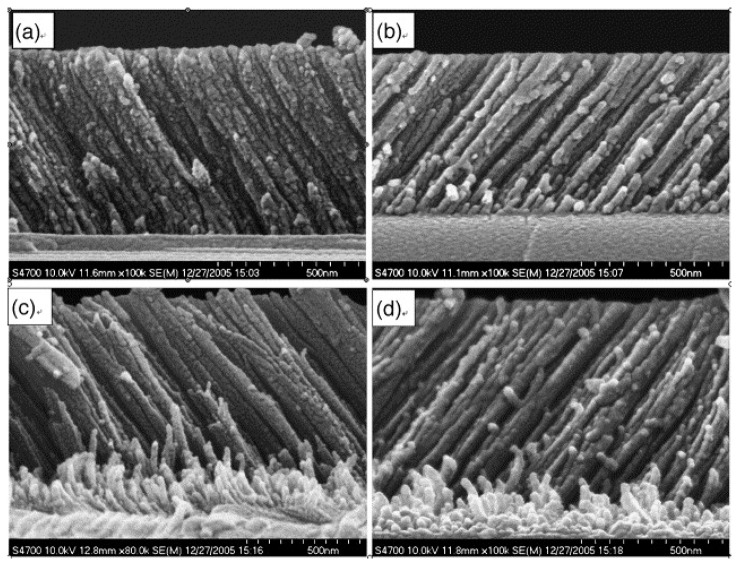
Cross-sectional SEM images of the thin TiO_2_ films deposited at different flux angles. (**a**) *α* = 60°; (**b**) *α* = 70°; (**c**) *α* = 75°; (**d**) *α* = 80°, reproduced from [31] with permission.

The trend to minimize the size of electronic devices reveals itself in the research and design of new humidity sensors [12,13]. Recently a new generation of electronic sensors based on CMOS-MEMS [7], nano-technology [14–18] and capacitive [19] technology has been reported in the scientific literature. These new sensors are based on nano-structured TiO_2_ thin films and have a reaction time which is approximately ten times shorter [19] than the devices that are widely used today. Figure 3 shows a nano-structured thin TiO_2_ film that was deposited using glancing angle deposition (GLAD).

Porous inorganic materials, such as porous silicon, have been investigated however organic materials have the potential for significantly lower cost. These can be prepared by low cost techniques such as spin coating, drop casting, dip coating or spray coating. Nickel phthalocynine (NiPc) and poly-*N*-epoxy-propyl-carbazole (PEPC) have been found to be sensitive to humidity. Ahmad *et al.* [20] have investigated the performance of mixtures of these compounds with copper oxide. The addition of copper oxide will increase the surface roughness to produce a larger surface area and has the potential to produce a greater sensitivity and reduced response time. Using this technique three times greater sensitivity was obtained however this material was only suitable for an operating range above 40% RH.

Graphene oxide is an interesting material for achieving a high sensitivity and fast response time due to its single layer nature and hydrophilic surface. Bi *et al.* [21] have investigated the use of these films as a humidity sensor. They improved the sensitivity by a factor of ten compared with conventional capacitance sensors and their sensor had a reasonable response time of 10.5 s for increasing humidity although this increased to 41 s for reducing humidity. The variation of the impedance of these structures with frequency was complicated and the devices only operated over a range of 15%–95% RH.

Multi-wall carbon nano-tubes (MWCNTs) [22] where the tube diameter varies are of interest for improving the performance of humidity sensors at low humidity due to capillary condensation. This is an effect where condensation can occur in thin capillaries at significantly below the saturation vapor concentration. The radius *r* required for condensation at a vapor pressure *p* is given by the Kelvin equation:

(1) $$r=-\frac{2\gamma V_{L}\cos(\Theta )}{\text{RTln}(\frac{p}{p_{0}})}$$

where *γ* is the surface tension, *V_L_* is the molar volume, Θ is the contact angle, *R* is the gas constant, *T* is the temperature, *p*_0_ is the saturation vapor pressure and $\frac{p}{p_{0}}$ is the relative humidity. The Kelvin radius *r* typically has a value of a few nanometers and increases as the RH increases. This allows more nanotubes to become filled with water. A sensor based on this principle was proposed by Chen *et al.* [22]. The sensor has improved sensitivity at low levels of humidity in comparison with a standard sensor without MWCNTs.

The relatively slow response of standard humidity sensors is a disadvantage in applications involving transient humidity changes such as in industrial process control and for monitoring atmospheric humidity. Kang and Wise [23] have developed a faster sensor based on polyimide that has a response time of around 1 s. Their design was based on an analysis of the diffusion into the sensor material. By etching the sensitive material into an array of pillars, they find that the increased surface area reduces the diffusion time by a factor often. By using pillars with diameters of 15, 10 and 5 *μ*m the response time was reduced to 6.9, 1.9 and 1.0 s respectively. A second feature of their design was to include an integrated poly-silicon heater and temperature sensor. This was used to prevent the temperature dropping below the dew point to maintain a short time of response and also to measure high humidity without having the sensor exposed to it. Using the heater, the relative humidity was calculated using the formula [23]:

(2) $$RH_{T=T_{1}}=\frac{p_{0}(T_{2})}{p_{0}(T_{1})}RH_{T=T_{2}}$$

where *T*_1_ and *T*_1_ are the temperatures before and after heating and *p*_0_(*T*) is the saturation pressure at a temperature *T*.

For a low-cost device, there is considerable advantage if the sensor material is compatible with silicon processing. Thin organic films fall into this category and several papers have described sensors compatible with MEMS [25,26] or CMOS [27,28] technology. The CMOS used top level metal and a standard passivation layer as the sensor. A self-calibration circuit was added to simplify the operation. MEMS technology allows multiple sensors (for temperature, humidity, pressure, wind speed and wind direction) to be integrated on a single chip.

### 2.3. Resistive Humidity Sensors

As with capacitive sensors, recent research on resistive sensors has been aimed at improving their sensitivity, response time, linearity and hysteresis characteristics.The characteristics of some recently reported resistive humidity sensors are listed in Table 3. Arshaka *et al.* have investigated the use of thermally deposited In_2_O_3_ [32] and sintered pastes of MnZn ferrite [33] and achieved low hysteresis and good linearity.

Kuang *et al.* [34] have investigated the properties of a single SnO_2_ nano-wire as a resistive humidity sensor. The use of a single nano-wire will lead to a very small sensor and may lead to a faster response time. The growth process resulted in a high concentration of oxygen vacancies leading to the surface of the nano-wire being very sensitive to oxygen and water vapor in the air. Characterization of this device showed a reproducible linear response with response and recovery times in the range 120–170 s and 20–60 s respectively. Lee [35] has researched the properties of nano-structured carbon nitride CN*_x_* films deposited by radio-frequency (RF) sputtering. The CN*_x_* bonds are expected to react reversibly with hydrogen and hydroxyl groups to generate a hydrophilic surface which can absorb and release water molecules. The resulting layers were found to have a reasonably linear response with a hysteresis which depended strongly on the substrate used. This was due to the formation of ink bottle shaped defects which trap water in the interior.

Polyimide films have been successfully used as capacitive humidity sensors however they have also been considered for use as resistive humidity sensors. Although the resistance of these devices is very sensitive to humidity, with the resistance changing by many orders of magnitude, they are not ideal humidity sensors due to their variability (requiring calibration), highly non-linear response and poor response at low humidity. Yoo *et al.* [17] have investigated a sensor that was manufactured by adding plasma-treated MWCNs to the polyimide film to improve its performance. As the doping level was increased, the resistance of the films remained very high until the density exceeded the percolation threshold of 0.05% where the resistance dropped rapidly. At sufficiently high concentrations, the resistance is governed by transport of electrons through the continuous paths, which the nano-tubes form. Increasing the humidity is found to increase the resistance due to a combination of charge transfer between the MWCNs and the water molecules, which reduces the mobile carrier concentration in MWCNs, and increased pressure due to swelling of the polyimide. For a nano-tube concentration of 0.4%, this leads to a highly linear variation of resistance with humidity.

**Figure 4.**
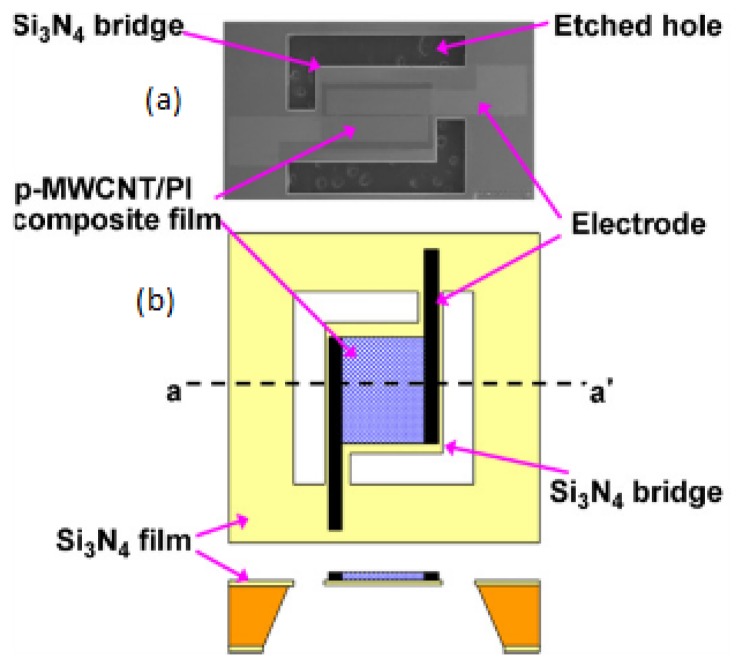
(**a**) Scanning electron microscope (SEM) image of active layer; and (**b**) schematic view of the resistive-type RH sensors based on p-MWCNT-PI composite film, reproduced from [17] with permission.

The extremely small size of these sensors drastically improves characteristics. The typical design of *p*-MWCNT/PI composite sensor is shown on Figure 4. Standard silicon micromachining was used to produce a thin film suspended over a silicon substrate. One major advantage of these sensors is their high linearity over the entire operational range. These sensors exhibit a sensitivity of 0.0047% RH.

## 3. Acoustic Humidity Sensors

In this section humidity sensors based on acoustic wave technology are reviewed. Acoustic methods of humidity measurements can be classified as mechanical methods. This measurement technique is based on the variation of mechanical properties of a hydrophilic material when water molecules are absorbed on to it. Existing methods of humidity measurement are based on surface acoustic waves (SAW), the change in the resonance frequency of a quartz crystal microbalance (QCM), quartz tuning forks (QTF) and on bulk acoustic waves (BAW) technology. The change in frequency of the acoustic resonance is typically used for detecting the change of material density that occurs because of absorption of water vapor from the surrounding atmosphere [39,40]. The change of density of the hydrophilic material changes the resonance frequency. This frequency shift has been theoretically analyzed by Sauerbrey [41]. The Sauerbrey equation gives the frequency shift of a quartz oscillator due to absorption of a small mass Δ*m* of water:

(3) $$\Delta f=-\frac{2f^{2}}{A\sqrt{\mu \rho }}\Delta m$$

where *f* is resonant frequency of the circuit, Δ*f* is the frequency change, Δ*m* is change of mass due to vapor absorption, *A* is the area of active crystal, *ρ* is quartz density (*ρ* = 2.648 g/cm^3^) and *μ* is Shear modulus of quartz for AT-cut crystal (*μ* = 2.947 × 10^11^ g/cm·s^2^).

This section is organized as follows: in the first subsection we provide a review of most relevant achievements in the branch of SAW sensors, the second subsection describes the state-of-art in the sector of quartz crystal microbalance humidity sensors. Although, there are not many publications devoted to investigating of BAW and QTF humidity sensors we have included a short description of advances in this area in the third subsection. Moreover, we have gathered together the most relevant characteristics of all these types of acoustic sensors in Tables 4 and 5.

### 3.1. Surface Acoustic Waves Humidity Sensors

Generally, SAW sensors are based either on organic molecules or on polymers whose interactions with the absorbed water molecules leads to a change in the velocity of surface waves. During the last decade experimental efforts have been focused on improving the surface quality and testing new hydrophilic materials. Various techniques such as drop coating [62], the Langmuir-Blodgett technique [63], spin coating [64], coating using the method of fast expansion of a supercritical solution (RESS) [65], electro-sprayed silicon-containing polyelectrolyte [66] and air-brush coating [67] have been considered for the deposition of the active material. For the electro-spray coating process, Li [44] has examined the influence of the polymer deposition rate and polymer solution concentration on the properties of the humidity sensor.

The effect of droplet diameter on intrinsic acoustic losses in the sensor have been investigated by Sarcar and co-workers [68]. Sarkar compared the coating film quality and performance of the sensor that was coated using the AC electro-spray technique with similar ones that were coated using the DC electro-spray technique, the air-brush and RESS. They concluded that much lower acoustic losses were obtained using the DC electro-spray process due to the rapid evaporation of the nano-droplets.

Very fast SAW sensors based either on thin films of poly-vinyl-alcohol (PVA) or on thin films of poly-vinyl-pyrrolidone (PVP) have been described by Buvailo [69]. These sensors show response/recovery times 1.5/2.5 s respectively in the humidity interval of 5%–95%.

### 3.2. Quartz Crystal Microbalance Humidity Sensors

These sensors tend to be quite slow in comparison with SAW sensors, with a response time of hundreds of seconds and quite non-linear behavior. However, some interesting findings have recently been published.

A hybrid sensor has been demonstrated by Shinbo [52]. This sensor was fabricated by depositing either slab or ridge optical waveguides made of fluorinated polyimides on the quartz crystal microbalance. Accurate discrimination of adsorbed chemical species has been successfully performed by observing the change in the mass load and either the optical transmittance or optical spectrum.

Su and co-workers [53] have compared the behavior of a sensor using composite of nafion with single-wall carbon nano-tubes (SWCNTs) with a sensor using multi-wall embedded carbon nano-tubes. This sensor showed quasi-linear behavior and a recovery time close to 100 s. Novel low-humidity sensors were fabricated *in situ* using photo-polymerization of polypyrrole nano-particles [54]. These sensors showed good sensitivity at low humidity and an unusually short response time. The authors associated this phenomenon with high local electrostatic charge of TiO_2_ nano-particles, which caused dissociation of water molecules.

In [55] it was shown that the sensitivity of fibrous composite polyacrylic acid (PAA)/polyvinyl-alcohol membranes was two times higher than the sensitivity of a corresponding flat film at 95% RH. The membranes based on fibrous composite have the highest sensitivity because of their extremely high surface area.

The use of zinc oxide has been investigated in [56,58,59]. These experiments have confirmed the well known fact [50] that the thickness of the sensitive film needs to be optimized in order to achieve the best possible performance. In addition, it was concluded that the structure of the zinc oxide film was important for sensor performance. Films structured in the form of either nano-rods or nano-wires showed slightly better performance than films structured in form of nano-tetrapods.

Finally, the hydrophilic potential of MWCNT [57] was investigated using measurements with QCM. The sensor, operating in the range between 5% and 95% of RH, has the response/recovery times of 60 and 70 s respectively and exhibits linear behavior.

### 3.3. Bulk Acoustic Waves and Quartz Tuning Forks Humidity Sensors

The resonant frequency of these devices strongly depends on temperature and research aimed at reducing the sensor temperature dependence is presented in [60]. This sensor takes advantage of the coexistence of fundamental and higher order resonance modes in the interval 30–100 MHz. The temperature dependence of the sensor has been successfully compensated in the range from 20% to 92% RH over the temperature range from 25 to 70 °C.

Zhou *et al.* [51] have presented the application of QTF coated with nano-crystalline ZnO film as a relative humidity sensor. Moreover, the interferences, which can be induced by either ethanol or acetone vapors have been discussed. The results shown that the uniformity of the ZnO film is important for the performance of the sensor.

## 4. Optical Humidity Sensors

The intensive investigation of the potential of optical fiber sensors began in the middle of the 1980s, after the first optical fibers became commercially available. At that time, the technology for electronic sensing was already well established. However, from the beginning, optical sensors have been successfully employed for a range of specific applications which electronic sensors are unable to perform. These include electromagnetic compatibility, multi-point measurements and the possibility of remote interrogation. Additional advantages of fibre sensors include miniature size and small weight. All these features make optical fibre sensors suitable for applications where electronic or acoustic ones are either not recommended or inappropriate. For example, fibre sensors offer a solution for monitoring parameters such as temperature and humidity inside microwave ovens. Optics sensors do not use electricity and consequently they can be used for monitoring of inflammable liquids or gases because of the absence of sparks. Fibre sensors are also a viable alternative in harsh environments such as those with corrosive substances [70]. Some of these sensors are also chemically inert, which allow their use in chemical reactors. Moreover, these sensors have been successfully used for monitoring historical objects in remote places [71]. For these reasons the acceptance of fibre sensors in several industrial sectors is growing steadily. Characteristics of some optical humidity sensors, which are commercially available today are summarized in in Table 6.

The research on optical humidity sensors started about 25 years ago. During the last 20 years the prices of fiber components have fallen and the quality of these components has been improved. The available range of fiber based sensors has significantly increased and can replace traditional sensors in many applications such as gyroscopes, tension and bending sensors, stress sensors, humidity and water level sensors.

For humidity sensing, a range of different techniques and approaches have been proposed including spectroscopy, change of reflectance of surfaces [72], evanescent wave interactions [73], Bragg and long period gratings [74–78], interferometers [79], carbon nano-tubes [14] and more recently, photonic crystal fibres [8,80]. Most fibre optic humidity sensors require a hygroscopic material, which is typically deposited on a section or on the tip of the optical fibre [81]. Humidity changes the optical properties of the material, which in turn, modifies a feature of the guided light giving rise to a detectable signal. Bedoya [82] has discussed an interesting fiber-optic humidity sensor based on the optical properties of indicator dyes.

In this section we will summarize the technological advancements that have recently emerged in the branch of sensors based on optical fiber technology. In the first subsection the latest achievements in the interferometric humidity sensing are reviewed. The second subsection is dedicated to the humidity sensors that are based on long period gratings. Advances in hybrid relative humidity sensors are illustrated in the third section. Finally, in the fourth and the fifth subsections the progress in humidity sensing based on evanescent-waves interactions and some other exotic humidity sensors are described. The main features of some optical fibre-based humidity sensors are summarized in Tables 7 and 8.

### 4.1. Interferometric Humidity Sensors

Interferometric techniques are the most exact and fastest of all the existing optical methods of measurement. Interferometric relative humidity sensing has been successfully tested in medicine [97] to monitor human breathing. This application in particular is useful for the diagnosis and study of the progression for such serious diseases as the sleep apnea syndrome. The most significant characteristics of humidity sensors based on interferometric measurements are their relative simplicity, extremely high sensitivity and short time of response. The main advantage of interferometric humidity sensing is the absence of hydrophilic material that can be easily damaged when the sensor works in a harsh environments [70]. The main disadvantage of these sensors is their low selectivity. This sort of sensors measures the changes in the refractive index of the atmosphere next to the fibre, consequently they are sensitive to a variety of contaminants. For this reason the interferometric techniques have sometimes been complemented by the use of hydrophilic materials. Numerous research groups have investigated diverse sensor architectures to improve sensor sensitivity and robustness.

The sensor based on the fiber Sagnac interferometer was developed by Chen [98]. The hydrophilic material called chitosan has been used in this sensor. Chitosan swells when the humidity is increased. This was used to produce strain and changes the polarization properties of the fiber. This sensor was compared with the sensor implemented in Fabry-Perot geometry using the same material [81]. The sensor based on Fabry-Perot geometry showed an extremely short response time of 380 ms.

**Figure 5.**
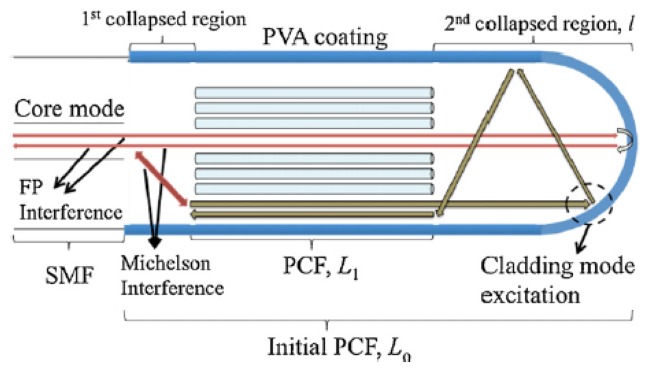
The sensor based on PVA coated photonic crystal interferometer, reproduced from [94] with permission.

A miniature optical RH sensor based on a polymer infiltrated photonic crystal fiber was reported by Mathew [99]. Experiments showed that the sensitivity of a sensor based on photonic crystal fiber can be improved [100] by infiltrating the voids of the photonic crystal with a hydroscopic polymer. At a later date, Wong [94] combined PVA, which is considered to be a very promising material for humidity sensing, with the interferometric technique proposed by Mathew [100]. The sensor is illustrated in Figure 5. The sensor with a 9% (*w*/*w*) coating achieved a sensitivity of 0.60 nm/% RH, exhibited little hysteresis, high repeatability, low cross-sensitivity to temperature and ammonia gas and stable performance during a seven day testing period. Moreover, the sensor showed short rise/fall times of 300/500 ms respectively.

Finally, Liang [101] investigated the combination of a PVA coating and loop mirror based on polarization maintaining fiber. His experiments showed quite a narrow range of sensitivity between 20% and 85% RH.

### 4.2. Humidity Sensors Based on Long Period Grating

**Figure 6.**
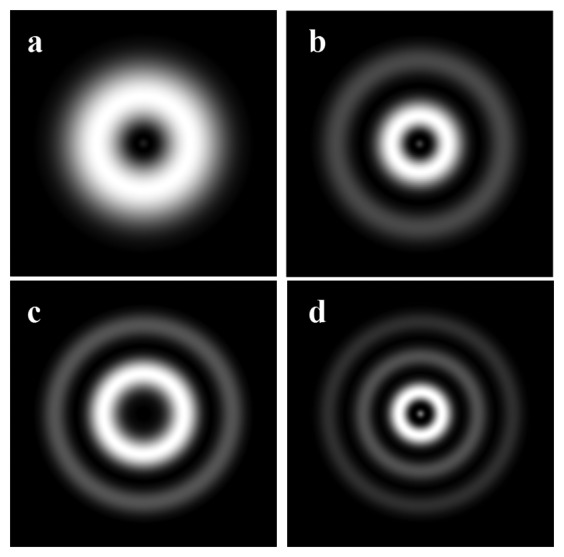
The intensity distribution of the first cladding modes (**a**) *l* = 1, *ν* = 1; (**b**) *l* = 1, *ν* = 3; (**c**) *l* = 1, *ν* = 4; (**d**) *l* = 1, *ν* = 5). The modes were simulated using [105].

This subsection will review research on humidity sensors based on long period fiber gratings (LPFG). In this kind of sensor a LPFG is used to excite cladding modes [102]. In Figure 6 the theoretical distribution of intensity of some cladding modes are illustrated. The experiential images of intensity distribution of *HE*_15_ and *HE*_16_ cladding modes can be find out, for example, in [103]. Cladding modes are extremely sensitive to changes in the refractive index of the material that surrounds the fiber. This property of the cladding modes is widely used for sensing applications. However, these sensors have some disadvantages. For example, Alwis [104] reported that the attenuation bands formed by the coupling between the propagation mode in the core and the cladding modes are very broad when an LPFG based sensor in transmission mode is used.

Often, the segment of fibre with the exited cladding modes is coated with some hydrophilic material. This improves the sensitivity and selectivity of the sensor so that it is mainly sensitive to humidity changes. Other fiber coatings, which have been investigated recently were based on thin films of calcium chloride [106], poly(ethylene oxide)/cobalt chloride (PEO/CoCl_2_) [107], diamond-like carbon [108], hydrogel [109,110], polyimide coating [111].

Venugopalan [112] reported LPFG with a PVA coating. Surprisingly, this sensor showed a relatively long response time of 50 s. The range of humidity sensing was only from 33% to 97% RH. In other types of humidity sensors this coating has achieved significantly better performance. Viegas [113] achieved an improvement in the sensitivity in a range 20% to 80% of a sensor, which was coated with a film of SiO_2_ nano-spheres using deposition through electrostatic self-assembly. As a result, the wavelength shift was increased from 5 to 15 nm.

### 4.3. Hybrid RH Sensors

Most of the sensors, that are available today provide humidity measurements in relative humidity units. At a constant pressure of gas the relative humidity will change if the temperature changes, even if the concentration of water vapor in the gas remains the same [114]. Therefore, the development of hybrid sensors that are capable measuring RH and temperature changes simultaneously is a necessary step to enable optical sensors to work over a range of temperatures for a variety of applications.

**Figure 7.**
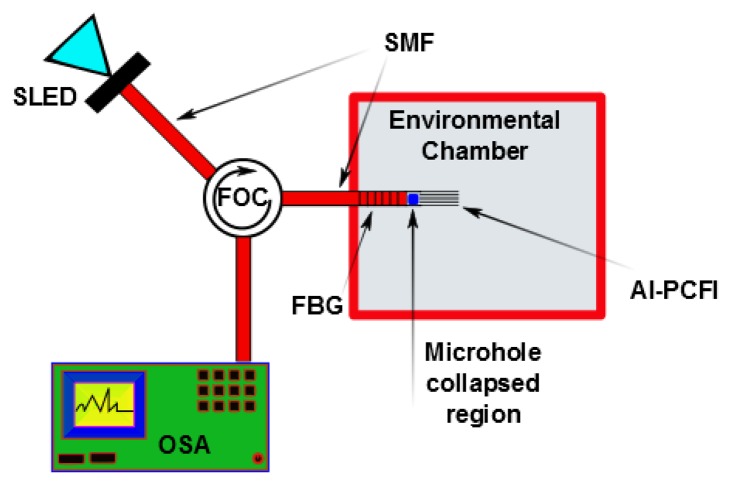
Schematic diagram of the hybrid fiber optic sensor system for simultaneous measurement of RH and temperature. (SLED—Super luminescent diode. FOC—Fiber optic circulator. SMF-Single mode fiber. FBG—Fiber Bragg grating. AI-PCFI—Agarose infiltrated-photonic crystal fiber interferometer. OSA—Optical spectrum analyzer. Dotted arrows represent the light path), adapted from [114].

A fast sensor with a linear response has been developed by Gu [79] using a thin-core fiber modal interferometer with a fiber Bragg grating written in the interior of the interferometer. Poly (N-ethyl-4-vinylpyridinium chloride) and salt of poly-vinyl-sulfonic acid and sodium were used as the hydrophilic material. The FBG was used to compensate for changes in the temperature. The implementation of the photonic crystal fibre interferometer [100] with FBG and using agarose as the hydrophilic material was proposed by Mathew *et al.* [114]. Figure 7 illustrates the scheme of the proposed sensor. This sensor produced a variation in the detected optical power of over 7 dB for a RH range of 75% and had a low level of temperature-humidity crosstalk. Other sensors based on a LPFGs include: a Mach-Zehnder interferometer based on cascaded long-period gratings coated with a thin-film of hydro-gel [110]; a Michelson interferometer using a LPFG grating pair formed by coating a mirror at the distal end of the LPFG [104]; and an LPFG using a tailored layered polyimide coating on the grating region [111] were put into operation. The former detector only operated over a limited (60%–100%) RH range. However, the second and third sensors showed operability over a range of 20%–80% RH, which was wider than the range where the former one operated.

### 4.4. Evanescent Wave Humidity Sensors

The principle of operation of evanescent field humidity sensors (EFHS) is quite simple. Electromagnetic field propagating inside the core of a waveguide do not confined completely inside it [115]. The fraction of the field that is confined inside of the core of a waveguide is called either the guided wave or the guided mode. In the same time, the part of the field that propagates outside of the waveguide is called either the evanescent wave or the evanescent field. The guided field and the evanescent field are related by condition of continuity of electromagnetic field on the border between the core and the cladding. This means that the guided field will be affected by any changes that the evanescent field experiences. When some water molecules are absorbed into the hydrophilic material the refractive index of the hydrophilic material changes. If the evanescent field propagates inside of the hydrophilic material, this change will affect the evanescent field and can be detected as a change in the guided mode. One of the common way to detect the changes in the evanescent field is, for example, by using an interferometer [79].

Today, efforts to improve of the performance of EFHS are mainly focused on research into novel, more sensitive hydrophilic materials as, for example, mats of electro-spun nano-fiber [116], layer of di-ureasil xerogel containing lithium bits [76,77], titanium dioxide nano-particles [117] or thin film of silica sol-gel [118]. In addition, different ways to generate evanescent field were tested, as for example, hetero-core optical fibers [119], no-core fiber structures [120], sub-wavelength diameter fiber taper [90] and multi-modal fibres [121].

Fuke [122] has published the results of synthesis of Ag-polyaniline nano-composite for application in a fiber-based humidity sensor using wave absorption spectroscopy. The sensor has been tested and optimized by varying the size of silver particles and the cladding length. The sensor has been operate over the range of humidity 5%–95% RH. It has been shown that a reduction in the size of the deposited particles leads to a dramatic improvement in the sensor sensitivity and the speed of response. A similar effect has also been observed for acoustic sensors. However even after optimization, the sensor had relatively long response time of 30 s and a recovery time of 90 s.

A humidity sensor based on a multi-mode fiber taper coated with polyvinyl alcohol has been investigated by Li [91]. The principle of operation of this sensor was based on the interaction between the optical evanescent field and the coating along the taper waist. This sensor showed maximum sensitivity of 1.994 *μ*W/% RH over the humidity range 30%–95% RH, fast response of 2 s and small temperature crosstalk.

Another interesting result has been published by Lui. In his experiments with regularly aligned nano-rods, Liu [123] has showed that a structure based on zinc oxide nano-rods radially grown on a silica fiber allows improved sensitivity of a sensor more than 50 times in comparison with the case of a fibre covered with disordered nano-particles of zinc oxide. The sensor showed good sensitivity over a wide range (10%–95% RH) of sensing.

### 4.5. Other Optical Humidity Sensors

**Figure 8.**
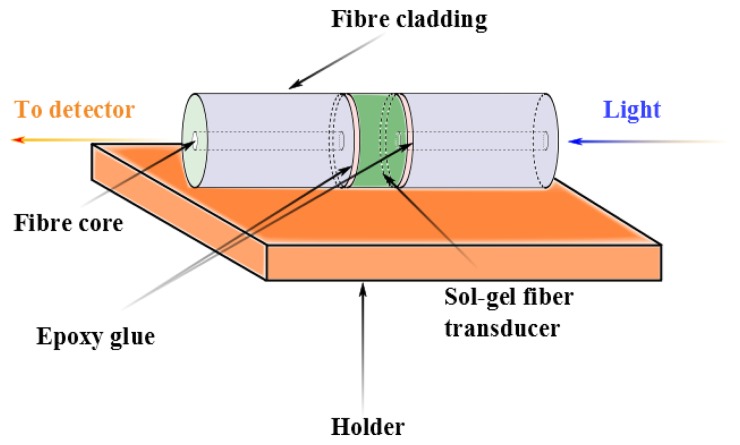
Example of an AFCOS sensor, adapted from [124].

A new concept in optical-fiber humidity sensor called either the active fiber core optical sensor or AFCOS, has been presented by Tao [124]. In this sensor, the fiber core plays the role of a transducer (see the example in Figure 8).

This type of sensor is based on a technique for doping porous sol-gel optical fibers with chemical reagents (CoCl_2_). These sensors are sensitive down to very low humidity levels (2% RH) but are not useful for an environment with higher than 10% RH humidity.

A simple, inexpensive optode for relative humidity (RH) monitoring in air has been fabricated by Bedoya [82] using the water-sensitive luminescent dye. The optode was able to measure humidity in the range from 4% to 100% RH, but had a very long response time of 1.4 min. The stability of the sensor was verified over 2.5 years for weather monitoring and measuring the humidity level in food. The sensor showed good stability and had the ability to recover completely after been soaked.

**Figure 9.**
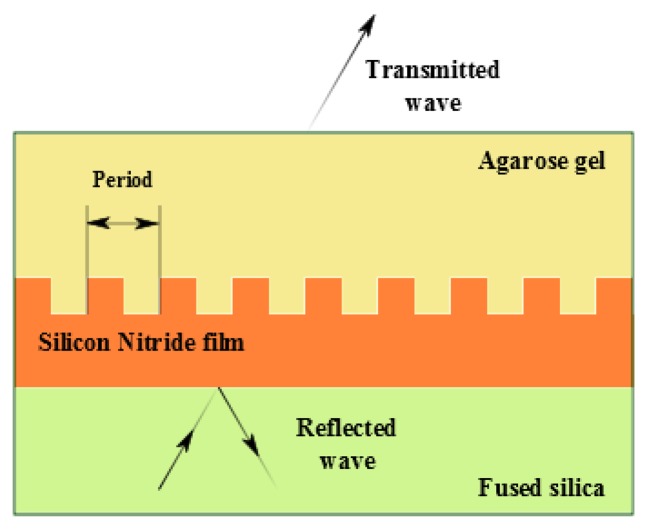
The guided-mode resonance sensor configuration, adapted from [125].

A non-fiber sensor has been developed by Lee and coworkers [125]. This sensor was based on changes in a guide-mode resonance using an agarose-gel transducer layer integrated with a periodic silicon-nitride film. The operation principle of the sensor is illustrated in Figure 9. The incident beam of light is phase matched to the periodic structure and is reflected along the layer of fused silica. The resonance wavelength changes when the hydrophilic material absorbs water. This change was used to detect humidity variations.

## 5. Conclusions

Today, the standard low-cost humidity sensors are the capacitance and resistance based electronic sensors. Significant advances have been made over the last ten years in addressing the limitations of these sensors and improving their ease of use. In particular, there has been progress in improving the speed of response to below 1 second and in developing sensors that are compatible with silicon technology. This compatibility has been exploited to allow integration with temperature and other sensors; generate an easy to use digital output; incorporate a heater which allows both the operation in atmosphere with the high humidity and either fast recovery from condensation or avoidance of it.

Today, a new generation of humidity sensing technology based on optical fibers is emerging. Humidity sensors based on the concept of the photonic crystal fiber are expected to be available soon. These sensors will be faster than the electronic humidity sensors that are available today. The current trends suggest that for many applications, electronic humidity sensors could soon either be replaced or complemented by sensors that are based on photonic crystals.

Furthermore, we highlight what we believe are two of the most significant achievements in the area of humidity sensing in the recent scientific literature. Polyvinyl alcohol (PVA) has emerged as a new and very promising material for humidity detection. This material allows fabrication of very fast and selective sensors with response times of less than one second. The second area which has seen significant advances is in the field of interferometric sensors. This novel sensor technology, presented by Gerald Farrell, Yuliya Semenova and Sunish Mathew from Dublin Institute of Technology, constitutes an important advance in sensing technology. It was demonstrated experimentally [99] that a simple design, using a laser diode as an interrogator is possible to use with this kind of sensors. Meanwhile, most of the sensors that are based on optical fibers require the use of a spectrum analyzer as the interrogator. The technology, which allows the use of low cost laser diodes as the interrogators, is expected to be attractive for industrial exploitation.

Today the first humidity sensors based on fiber optics are starting to appearing on the markets. We expect that the application of fiber-based sensors will grow exponentially throughout the next decade. Initially, optical humidity sensors will satisfy specific unfulfilled applications in the chemical industry, where humidity control would be beneficial but present electronic sensors cannot satisfy the need. There are applications in the chemical industry, where there is the need to monitor tanks with corrosive, flammable or explosive atmospheres, and where fiber optic humidity sensors, such as those based on the interferometric technique or fiber Bragg gratings could offer a unique solution. Another attractive feature of the fiber based technology is the relative ease of integrating centralized remote monitoring and control over a number of separate facilities related with the low cost and low weight of the optical fiber cable in comparison with a copper cable . An optical interrogation module can be designed to allow simultaneous interrogation of tens or even hundreds of sensors. This can be installed in a remote office allowing the operator to monitor a set of sensors covering an area of up to a few miles in radius. In addition, recent scientific advances should allow lower cost dedicated systems by avoiding the relatively high price of interrogation modules which are presently a significant disadvantage of fiber-based sensors.

## Figures and Tables

**Table 1.** Performance of commercial electronic humidity sensors.

**Sensor**	**Type**	**Response, s**		**Range, %RH**		**Hysteresis, %**

		**Inc**	**Dec**	**min**	**max**	
SHT15 Polymer	C	8	8	0	100	2
DHT22	C	NA	NA	0	100	2
HR202	R	10	10	20	95	1
DHT11	R	10	10	20	90	4
HMT330 ^1^	Electronic	8	17	0	100	1
HMT330 ^2^	Electronic	20	50	0	100	1
HMT330 ^3^	Electronic	40	60	0	100	1
HC2-C	Electronic	15	NA	0	100	NA
Fluke 971	Electronic	60	NA	5	95	NA

1. With grid filter;

2. With steel netting filter;

3. With sintered filter.

**Table 2.** Performance of capacitative humidity sensors.

**Sensor**	**Type**	**Response, s**		**Range, %RH**		**Hysteresis, %**	**Ref.**
	
		**Inc**	**Dec**	**min**	**max**		
Composite (PEPC + NiPc + Cu_2_O)	C	13	15	40	100	13	[20]
Graphene oxide film	C	10.5	41	15	95	5	[21]
MWCNTs	C	45	15	11	97	NA	[22]
Polyimide film	C	1	1	30	90	2	[23]
3 *μ*m polyimide film on parylene on silicon	C	1	1	30	90	2	[24]
Polyimide film	C	1.7	2.3	30	100	NA	[25]
Polyimide film	C	NA	NA	50	90	NA	[26]
Standard CMOS polymer film	C	70	70	10	95	5.5	[27]
Standard CMOS polymer film	C	70	70	10	95	3.1	[28]
Polypyrrole	C	NA	NA	25	95	small	[29]
Mesoporous silica	C	NA	NA	0	50	NA	[30]
Capacitive-dependent crystal	C	0.1	0.1	20	98	0.3	[12,13]

**Table 3.** Performance of resistive humidity sensors.

**Sensor**	**Type**	**Response, s**		**Range, %RH**		**Hysteresis, %**	**Ref.**
	
		**Inc**	**Dec**	**min**	**max**		
In_2_O_3_/SiO	R	NA	NA	40	90	NA	[32]
MnZn Ferrite	R	17	25	30	90	0.97	[33]
Fe-Al-polyaniline on CMOS	R	NA	NA	32	55	NA	[36]
SnO_2_ nanowire	R	120–170	20–60	5	85	NA	[34]
CN*_x_* deposited by RF sputtering	R	150	175	5	95	3	[35]
Sulfonated Polycarbonate	R	NA	NA	11	90	4	[37]
Polyaniline/PVA	R	NA	NA	25	85	NA	[38]
MWCNT/polyimide composite film	R	NA	NA	10	95	NA	[17]

**Table 4.** Performance of acoustic humidity sensors.

**Sensor**	**f**	**Sensitivity**	**Response**	**PH (%)**		**Ref.**

	**(MHz)**	**(ppm^7^/%RH)**	**Time (s)**	**min**	**max**	
MWCNT/Nafion nanofiber film ^1^	∼500	885.2	10	10	80	[42]
APTS-P ^1^ [43]	433	15	10	10	100	[44]
PVA-coated dual resonator ^1^	∼468	11–395	1,800	20	80	[45]
Cellulose acetate film ^1^	302	3.31	NA	10	80	[46]
Nafion layer ^1^	∼302	2.75	≤20	10	100	[47,48]
NPF-LSAW ^1^	60–110	0–6 ^6^	0.25	10	90	[49]
Monodisperse mesoporous silica ^1^	10	250–720	10	10	100	[50]
Nanocrystalline Zn oxide ^2^	0.032	1.1597	≤150	10	100	[51]
Fluorinated polyimides ^3^	5	2	300	15	85	[52]
Nano-tubes/Nafion composite ^3^	9	0.1123 ^5^	100	23.4 ^5^	3,030.5 ^4^	[53]
Polypyrrole AgTiO_2_ nano-part. ^3^	10	0.0246 ^5^	12	0 ^5^	10,000 ^5^	[54]
Nanofibrous membrane ^3^	5	0.1–10	80–150	20	95	[55]
ZnO nano-tetrapods ^3^	10	0–3.3	NA	30	90	[56]
Carbon nano-tube film ^3^	25	≤4	60/70 ^8^	5	97	[57]
ZnO nanostructure film ^3^	25	0.26	90/120 ^8^	5	97	[58]
Nanostructured ZnO ^3^	10	≤1	NA	20	90	[59]
ZnO on silicon base ^4^	35/86	400/16	≤25	20	92	[60]
ZnO-FBAR ^4^	1,430	1.4	NA	25	85	[61]

1. SAW configuration;

2. Quartz tuning fork;

3. Quartz crystal microbalance humidity sensors;

4. Bulk humidity sensors;

5. In Hz/ppm_v;_

6. dB/PH(%);

7. Parts per million;

8. Response/recovery time.

**Table 5.** Linearity and hysteresis of acoustic humidity sensors.

**Sensor**	**Linearity**	**Hysteresis ^5^**	**Ref.**
MWCNT/Nafion nanofiber film ^1^	Linear between 10% and 75%	≤10%	[42]
APTS-P ^1^ [43]	Linear	NA	[44]
PVA-coated dual resonator SAW ^1^	Non linear	NA	[45]
Cellulose acetate film ^1^	Linear between 10% and 60%	≤7%	[46]
Nafion layer ^1^	Linear between 10% and 60%	≤12%	[47,48]
NPF-LSAW ^1^	Non linear	NA	[49]
Monodisperse mesoporous silica ^1^	Linear between 0% and 80%	≤5	[50]
Nanocrystalline Zn oxide ^2^	Linear between 40% and 100%	NA	[51]
Fluorinated polyimides ^3^	Non linear	NA	[52]
Carbon nano-tubes/Nafion composite ^3^	Linear	NA	[53]
Polypyrrole AgTiO_2_ nano-particles ^3^	Linear	NA	[54]
Nanofibrous membrane ^3^	Exponential between 20% and 95%	NA	[55]
ZnO nano-tetrapods ^3^	Non linear	NA	[56]
Carbon nano-tube film ^3^	Linear	NA	[57]
ZnO nanostructure film ^3^	Linear	NA	[58]
Nanostructured ZnO ^3^	Non linear	NA	[59]
ZnO on silicon base ^4^	Non linear	NA	[60]
ZnO-FBAR ^4^	Non linear	NA	[61]

1. SAW configuration;

2. Quartz tuning fork;

3. Quartz crystal microbalance humidity sensors;

4. Bulk humidity sensors;

5. The width of hysteresis in relation to whole measured interval.

**Table 6.** Performance of commercial optic humidity sensors.

**Sensor**	**Accuracy**	**Response, s**	**Range, %RH**	

			**min**	**max**
Nanosonic Inc.	6% at 20 °C	0.1	0	100
O-eland FBG-based	4.5%	fast	10	100

**Table 7.** Performance of optic humidity sensors.

**Sensor**	**Sensitivity**	**Response, s**		**Range PH (%)**		**Ref.**

		**Inc**	**Dec**	**min**	**max**	
ZnO nanoparticles in solgel ^1^	0.0103 dB/%RH	0.86	0.54	5	95	[83]
SiO_2_ Nanoparticles ^1^	0.3 dB/%RH	0.15	0.1	40	98	[84]
Silica xerogel film ^1^	7.9 × 10 ^−2^ *nm*^−1^ · % ^−1^	10	120	4	100	[85]
Hollow core fiber ^1^	3.02 *mV*/1%RH	60	NA	0	90	[86]
Surface Plasmon Resonance ^1^	0.943 nm per RH%	<0.5	NA	5	95	[87]
CoCl_2_ into a PVA composite ^1^	0.5 dB/%RH	120	NA	25	65	[88]
Di-ureasil coated FBG ^2^	22.2 pm/%RH	600	NA	15	95	[77]
Bent single mode optical fiber ^2^	<0.1 dB/%RH	0.05	NA	25	90	[89]
Gelatin film ^2^	<0.1 dB/%RH	0.07	NA	9	94	[90]
Polyimide-coated FBG ^3^	^−^0.000266 V/%RH	5	NA	11	98	[75]
PVA coated FBG ^3^	1.994 *μ*W/%RH	2	NA	30	95	[91]
Tilted FBG with PVA ^3^	14.947 dBm/%RH	2	NA	20	98	[92]
Polyimide coated FBG ^3^	5.6 pm/%RH	2,700	NA	20	98	[93]
PVA ^4^	0.60 nm/%RH	3 × 10^−4^	5 × 10^−4^	30	90	[94]
FBG in a polymer fibre	35.2 pm/%RH	1,800	NA	50	95	[95]
Luminescent Ru(II) complex	NA	<90	NA	4	100	[82]
SiO_2_ nanospheres ^5^	0.2 nm/%RH	<0.02	NA	20	80	[96]

1. Absorption measurements;

2. Evanescent wave;

3. Strain;

4. Interferometric;

5. LPFG.

**Table 8.** Linearity and hysteresis of optic humidity sensors.

**Sensor**	**Linearity**	**Hysteresis^6^**	**Ref**.
ZnO nanoparticles in solgel ^1^	Linear	NA	[83]
SiO_2_ Nanoparticles ^1^	Linear 40%–98%	0.25%	[84]
Silica xerogel film ^1^	Linear 0%–60%	NA	[85]
Hollow core fiber ^1^	Linear	NA	[86]
Surface Plasmon Resonance ^1^	Linear	NA	[87]
CoCl_2_ into a PVA composite ^1^	Non linear	<3%	[88]
Di-ureasil coated FBG ^2^	Non linear	NA	[77]
Bent single mode optical fiber ^2^	Linear 25%–90%	NA	[89]
Gelatin film ^2^	Non linear	NA	[90]
Polyimide-coated FBG ^3^	Linear	NA	[75]
PVA coated FBG ^3^	Non linear	<4%	[91]
Tilted FBG with PVA ^3^	Linear 20%–80%	<10%	[92]
Polyimide coated FBG ^3^	Linear	<5%	[93]
PVA ^4^	Non linear	15%	[94]
FBG in a polymer fibre	Linear	NA	[95]
Luminescent Ru(II) complex	Non linear	4%	[82]
SiO_2_ nanospheres ^5^	Non linear	NA	[96]

1. Absorption measurements;

2. Evanescent wave;

3. Strain;

4. Interferometric;

5. long period fiber gratings (LPFG);

6. The width of hysteresis in relation to whole measured interval.

## References

[1] Tatara T., Tsuzaki K. (1997). An apnea monitor using a rapid-response hygrometer. J. Clin. Monit. Comput..

[2] Lin Y.C. (2013). Breath sensor based on reflective optical lensed fiber. Microw. Opt. Technol. Lett..

[3] Kastner W., Neugschwandtner G., Soucek S., Newmann H.M. (2005). Communication systems for building automation and control. Proc. IEEE.

[4] Laville C., Pellet C. (2002). Interdigitated humidity sensors for a portable clinical microsystem. IEEE Trans. Biomed. Eng..

[5] Habib Ahsan A.H.M., Lange C.F., Moussa W. Development of a Humidity Microsensor with Thermal Reset.

[6] Chairperson Publications Board (2008). Guide to Meteorological Instruments and Methods of Observation.

[7] Lazarus N., Fedder G.K. (2012). Designing a robust high-speed CMOS-MEMS capacitive humidity sensor. J. Micromech. Microeng..

[8] Mathew J., Semenova Y., Rajan G., Farrell G. (2010). Humidity sensor based on a photonic crystal fibre interferometer. Electron. Lett..

[9] Favero F., Villatoro J., Pruneri V. (2012). Microstructured optical fiber interferometric breathing sensor. J. Biomed. Opt..

[10] Zheng S., Zhu Y., Krishnaswamy S. (2013). Fiber humidity sensors with high sensitivity and selectivity based on interior nanofilm-coated photonic crystal fiber long-period gratings. Sens. Actuators B Chem..

[11] Cardenas-Sevilla G., Favero F., Villatoro J. (2013). High-visibility photonic crystal fiber interferometer as multifunctional sensor. Sensors.

[12] Matko V., Koprivnikar J. (1998). Quartz sensor for water absorption measurement in glass-fiber resins. Instrum. Meas. IEEE Trans..

[13] Matko V., Donlagic D. Sensor for High-Air-Humidity Measurement.

[14] Zhang T., Mubeen S., Myung N.V., Deshusses M.A. (2008). Recent progress in carbon nanotube-based gas sensors. Nanotechnology.

[15] Li H., Zhang J., Tao B., Wan L., Gong W. (2009). Investigation of capacitive humidity sensing behavior of silicon nanowires. Phys. E Low-Dimens. Syst. Nanostruct..

[16] Kauffman D.R., Star A. (2008). Carbon nanotube gas and vapor sensors. Angew. Chem. Int. Ed..

[17] Yoo K.P., Lim L.T., Min N.K., Lee M.J., Lee C.J., Park C.W. (2010). Novel resistive-type humidity sensor based on multiwall carbon nanotube/polyimide composite films. Sens. Actuators B Chem..

[18] Yeow J.T.W., She J.P.M. (2006). Carbon nanotube-enhanced capillary condensation for a capacitive humidity sensor. Nanotechnology.

[19] Steele J., Fitzpatrick G., Brett M.J. (2007). Capacitive humidity sensors with high sensitivity and subsecond response times. IEEE Sens. J..

[20] Ahmad Z., Zafar Q., Sulaiman K., Akram R., Karimov K.S. (2013). A humidity sensing organic-inorganic composite for environmental monitoring. Sensors.

[21] Bi H., Yin K., Xie X., Ji J., Wan S., Sun L., Terrones M., Dresselhaus M.S. (2013). Ultrahigh humidity sensitivity of graphene oxide. Sci. Rep..

[22] Chen W.P., Zhao Z.G., Liu X.W., Zhang Z.X., Suo C.G. (2009). A capacitive humidity sensor based on multi-wall carbon nanotubes (MWCNTs). Sensors.

[23] Kang U., Wise K. (2000). A high-speed capacitive humidity sensor with on-chip thermal reset. IEEE Trans. Electron Devices.

[24] Kuo L.S., Huang H.H., Yang C.H., Chen P.H. (2011). Real-time remote monitoring of temperature and humidity within a proton exchange membrane fuel cell using flexible sensors. Sensors.

[25] Lee C.Y., Su A., Liu Y.C., Chan P.C., Lin C.H. (2010). Sensor fabrication method for *in situ* temperature and humidity monitoring of light emitting diodes. Sensors.

[26] Ma R.H., Wang Y.H., Lee C.Y. (2011). Wireless remote weather monitoring system based on MEMS technologies. Sensors.

[27] Nizhnik O., Higuchi K., Maenaka K. (2011). A standard CMOS humidity sensor without post-processing. Sensors.

[28] Nizhnik O., Higuchi K., Maenaka K. (2012). Self-calibrated humidity sensor in CMOS without post-processing. Sensors.

[29] Yang M.Z., Dai C.L., Lu D.H. (2010). Polypyrrole porous micro humidity sensor integrated with a ring oscillator circuit on chip. Sensors.

[30] Wagner T., Krotzky S., Weiss A., Sauerwald T., Kohl C.D., Roggenbuck J., Tiemann M. (2011). A high temperature capacitive humidity sensor based on mesoporous silica. Sensors.

[31] Wang S., Xia G., He H., Yi K., Shao J., Fan Z. (2007). Structural and optical properties of nanostructured TiO_2_ thin films fabricated by glancing angle deposition. J. Alloy. Compd..

[32] Arshaka K., Twomey K. (2002). Thin films of In_2_O_3_/SiO for humidity sensing applications. Sensors.

[33] Arshaka K., Twomey K., Egan D. (2002). A ceramic thick film humidity sensor based on MnZn ferrite. Sensors.

[34] Kuang Q., Lao C., Wang Z.L., Xie Z., Zheng L. (2007). High-sensitivity humidity sensor based on a single SnO_2_ nanowire. J. Am. Chem. Soc..

[35] Lee S.P. (2008). Synthesis and characterization of carbon nitride films for micro humidity sensors. Sensors.

[36] Huang C.W., Huang Y.J., Lu S.S., Lin C.T. (2012). A fully integrated humidity sensor system-on-chip fabricated by micro-stamping technology. Sensors.

[37] Rubinger C.P.L., Calado H.D.R., Rubinger R.M., Oliveira H., Donnici C.L. (2013). Characterization of a sulfonated polycarbonate resistive humidity sensor. Sensors.

[38] Yang M.Z., Dai C.L., Lin W.Y. (2011). Fabrication and characterization of polyaniline/PVA humidity microsensors. Sensors.

[39] Penza M., Anisimkin V.I. (1999). Surface acoustic wave humidity sensor using polyvinyl-alcohol film. Sens. Actuators A Phys..

[40] Caliendo C., Verona E., Anisimkin V.I. (1997). Surface acoustic wave humidity sensors: a comparison between different types of sensitive membrane. Smart Mater. Struct..

[41] Sauerbrey G. (1959). The use of quartz oscillators for weighing thin layers and for microweighing (In German). Zeitschrift für Physik.

[42] Sheng L., Chen D., Chen Y. (2011). A surface acoustic wave humidity sensor with high sensitivity based on electrospun MWCNT/Nafion nanofiber films. Nanotechnology.

[43] Lv X., Li Y., Hong L., Luo D., Yang M. (2007). A highly water-resistive humidity sensor based on silicon-containing polyelectrolytes prepared by one-pot method. Sens. Actuators B Chem..

[44] Li Y., Li P., Yang M., Lei S., Chen Y., Guo X. (2010). A surface acoustic wave humidity sensor based on electrosprayed silicon-containing polyelectrolyte. Sens. Actuators B Chem..

[45] Penza M., Cassano G. (2000). Relative humidity sensing by PVA-coated dual resonator SAW oscillator. Sens Actuators B Chem..

[46] Braga E., Nakano A., da Cunha M. A SAW Resonator Sensor System Employed in Humidity Measurements.

[47] Kawalec A., Pasternak M. (2008). A new high-frequency surface acoustic wave sensor for humidity measurement. Instrum. Meas. IEEE Trans..

[48] Kawalec A., Jasek K., Pasternak M. (2008). Measurements results of SAW humidity sensor with nafion layer. Eur. Phys. J. Spec. Top..

[49] Rimeika R., Ciplys D., Poderys V., Rotomskis R., Shur M. Humidity Sensor Using Leaky Surface Acoustic Waves in YX-LiTaO_3_ with Nanostructured Porphyrin Film.

[50] Zhu Y., Yuan H., Xu J., Xu P., Pan Q. (2010). Highly stable and sensitive humidity sensors based on quartz crystal microbalance coated with hexagonal lamelliform monodisperse mesoporous silica SBA-15 thin film. Sens. Actuators B Chem..

[51] Zhou X., Jiang T., Zhang J., Wang X., Zhu Z. (2007). Humidity sensor based on quartz tuning fork coated with solgel-derived nanocrystalline zinc oxide thin film. Sens. Actuators B Chem..

[52] Shinbo K., Otuki S., Kanbayashi Y., Ohdaira Y., Baba A., Kato K., Kaneko F., Miyadera N. (2009). A hybrid humidity sensor using optical waveguides on a quartz crystal microbalance. Thin Solid Films.

[53] Su P.G., Sun Y.L., Lin C.C. (2006). A low humidity sensor made of quartz crystal microbalance coated with multi-walled carbon nanotubes/Nafion composite material films. Sens. Actuators B Chem..

[54] Su P.G., Chang Y.P. (2008). Low-humidity sensor based on a quartz-crystal microbalance coated with polypyrrole/Ag/TiO_2_ nanoparticles composite thin films. Sens. Actuators B Chem..

[55] Wang X., Ding B., Yu J., Wang M., Pan F. (2010). A highly sensitive humidity sensor based on a nanofibrous membrane coated quartz crystal microbalance. Nanotechnology.

[56] Wang X.H., Ding Y.F., Zhang J., Zhu Z.Q., You S.Z., Chen S.Q., Zhu J. (2006). Humidity sensitive properties of ZnO nanotetrapods investigated by a quartz crystal microbalance. Sens. Actuators B Chem..

[57] Zhang Y., Yu K., Xu R., Jiang D., Luo L., Zhu Z. (2005). Quartz crystal microbalance coated with carbon nanotube films used as humidity sensor. Sens. Actuators A Phys..

[58] Zhang Y., Yu K., Ouyang S., Luo L., Hu H., Zhang Q., Zhu Z. (2005). Detection of humidity based on quartz crystal microbalance coated with ZnO nanostructure films. Phys. B Condens. Matter..

[59] Zhou X., Zhang J., Jiang T., Wang X., Zhu Z. (2007). Humidity detection by nanostructured ZnO: A wireless quartz crystal microbalance investigation. Sens. Actuators A Phys..

[60] Fu J., Ayazi F. Dual-Mode Piezo-on-Silicon Resonant Temperature and Humidity Sensor for Portable Air Quality Monitoring Systems.

[61] Qiu X., Wang Z., Zhu J., Oiler J., Tang R., Yu C., Yu H. (2010). The effects of relative humidity and reducing gases on the temperature coefficient of resonant frequency of ZnO based film bulk acoustic wave resonator. IEEE Trans. Ultrason. Ferroelectr. Freq. Control.

[62] Tashtoush N.M., Cheeke J.D.N., Eddy N. (1998). Surface acoustic wave humidity sensor based on a thin PolyXIO film. Sens. Actuators B Chem..

[63] Li Y., Yang M.J., Ling M.F., Zhu Y.H. (2007). Surface acoustic wave humidity sensors based on poly(p-diethynylbenzene) and sodium poly sulfone sulfonate. Sens. Actuators B Chem..

[64] Penza M., Cassano G., Sergi A., Sterzo C.L., Russo M.V. (2001). SAW chemical sensing using poly-ynes and organometallic polymer films. Sens. Actuators B Chem..

[65] Levit N., Pestov D., Tepper G. (2002). High surface area polymer coatings for SAW-based chemical sensor applications. Sens. Actuators B Chem..

[66] Li Y., Li P., Yanga M., Lei S., Chenb Y., Guoc X. (2010). A surface acoustic wave humidity sensor based on electrosprayed silicon-containing polyelectrolyte. Sens. Actuators B Chem..

[67] Shen C.Y., Huang C.P., Huang W. (2004). Gas-detecting properties of surface acoustic wave ammonia sensors. Sens. Actuators B Chem..

[68] Sarkar S., Levit N., Tepper G. (2006). Deposition of polymer coatings onto SAW resonators using AC electrospray. Sens. Actuators B Chem..

[69] Buvailo A., Xing Y., Hines J., Borguet E. (2011). Thin polymer film based rapid surface acoustic wave humidity sensors. Sens. Actuators B Chem..

[70] Consales M., Buosciolo A., Cutolo A., Breglio G., Irace A., Buontempo S., Petagna P., Giordano M., Cusano A. (2011). Fiber optic humidity sensors for high-energy physics applications at CERN. Sens. Actuators B Chem..

[71] Garcia-Diego F.J., Fernandez-Navajas A., Beltran P., Merello P. (2013). Study of the effect of the strategy of heating on the mudejar church of Santa Maria in Ateca (Spain) for preventive conservation of the altarpiece surroundings. Sensors.

[72] Lin Y.C. (2013). Breath sensor based on reflective optical lensed fiber. Microw. Opt. Technol. Lett..

[73] Xu L., Fanguy J.C., Soni K., Tao S. (2004). Optical fiber humidity sensor based on evanescent-wavescattering. Opt. Lett..

[74] Yeo T., Sun T., Grattan K., Parry D., Lade R., Powell B. (2005). Polymer-coated fiber Bragg grating for relative humidity sensing. IEEE Sens. J..

[75] Huang X., Sheng D., Cen K., Zhou H. (2007). Low-cost relative humidity sensor based on thermoplastic polyimide-coated fiber Bragg grating. Sens. Actuators B Chem..

[76] Barbosa P.C., Silva M.M., Smith M.J., Gonçalves A., Fortunato E., Nunes S.C., de Zea Bermudez V. (2009). Di-ureasil xerogels containing lithium bis(trifluoromethanesulfonyl)imide for application in solid-state electrochromic devices. Electrochim. Acta.

[77] Correia S.F., Antunes P., Pecoraro E., Lima P.P., Varum H., Carlos L.D., Ferreira R.A., Andre P.S. (2012). Optical fiber relative humidity sensor based on a FBG with a di-ureasil coating. Sensors.

[78] Berruti G., Consales M., Giordano M., Sansone L., Petagna P., Buontempo S., Breglio G., Cusano A. (2013). Radiation hard humidity sensors for high energy physics applications using polyimide-coated fiber Bragg gratings sensors. Sens. Actuators B Chem..

[79] Gu B., Yin M., Zhang A.P., Qian J., He S. (2011). Optical fiber relative humidity sensor based on FBG incorporated thin-core fiber modal interferometer. Opt. Express.

[80] Mathew J., Semenova Y., Farrell G. (2012). Relative humidity sensor based on an agarose-infiltrated photonic crystal fiber interferometer. IEEE J. Sel. Top. Quantum Electron..

[81] Chen L.H., Li T., Chan C.C., Menon R., Balamurali P., Shaillender M., Neu B., Ang X.M., Zu P., Wong W.C. (2012). Chitosan based fiber-optic Fabry-Perot humidity sensor. Sens. Actuators B Chem..

[82] Bedoya M., Dez M.T., Moreno-Bondi M.C., Orellana G. (2006). Humidity sensing with a luminescent Ru(II) complex and phase-sensitive detection. Sens. Actuators B Chem..

[83] Aneesh R., Khijwania S.K. (2013). Zinc oxide nanoparticle-doped nanoporous solgel fiber as a humidity sensor with enhanced sensitivity and large linear dynamic range. Appl. Opt..

[84] Corres J., Matias I., Hernaez M., Bravo J., Arregui F. (2008). Optical fiber humidity sensors using nanostructured coatings of SiO_2_ nanoparticles. IEEE Sens. J..

[85] Estella J., de Vicente P., Echeverra J.C., Garrido J.J. (2010). A fibre-optic humidity sensor based on a porous silica xerogel film as the sensing element. Sens. Actuators B Chem..

[86] Noor M.Y.M., Khalili N., Skinner I., Peng G.D. (2012). Optical relative humidity sensor based on a hollow core-photonic bandgap fiber. Meas. Sci. Technol..

[87] Rivero P.J., Urrutia A., Goicoechea J., Arregui F. (2012). Optical fiber humidity sensors based on Localized Surface Plasmon Resonance (LSPR) and Lossy-mode resonance (LMR) in overlays loaded with silver nanoparticles. Sens. Actuators B Chem..

[88] Wang B., Zhang F., Pang F., Wang T. An Optical Fiber Humidity Sensor Based on Optical Absorption.

[89] Mathew J., Semenova Y., Farrell G. (2012). A fiber bend based humidity sensor with a wide linear range and fast measurement speed. Sens. Actuators A Phys..

[90] Zhang L., Gu F., Lou J., Yin X., Tong L. (2008). Fast detection of humidity with a subwavelength-diameter fiber taper coated with gelatin film. Opt. Express.

[91] Li T., Dong X., Chan C.C., Zhao C.L., Zu P. (2012). Humidity sensor based on a multimode-fiber taper coated With polyvinyl alcohol interacting with a fiber bragg grating. IEEE Sens. J..

[92] Miao Y., Liu B., Zhang H., Li Y., Zhou H., Sun H., Zhang W., Zhao Q. (2009). Relative humidity sensor based on tilted fiber bragg grating with polyvinyl alcohol coating. IEEE Photonics Technol. Lett..

[93] Yeo T.L., Sun T., Grattan K.T.V., Parry D., Lade R., Powell B.D. (2005). Characterisation of a polymer-coated fibre Bragg grating sensor for relative humidity sensing. Sens. Actuators B Chem..

[94] Wong W.C., Chan C.C., Chen L.H., Li T., Lee K.X., Leong K.C. (2012). Polyvinyl alcohol coated photonic crystal optical fiber sensor for humidity measurement. Sens. Actuators B Chem..

[95] Zhang C., Zhang W., Webb D.J., Peng G.D. (2010). Optical fibre temperature and humidity sensor. Electron. Lett..

[96] Viegas D., Goicoechea J., Corres J.M., Santos J.L., Ferreira L.A., Araújo F.M., Matias I.R. (2009). A fibre optic humidity sensor based on a long-period fibre grating coated with a thin film of SiO_2_ nanospheres. Meas. Sci. Technol..

[97] Favero F.C., Villatoro J., Pruneri V. (2012). Microstructured optical fiber interferometric breathing sensor. J. Biomed. Opt..

[98] Chen L.H., Chan C.C., Li T., Shaillender M., Neu B., Balamurali P., Menon R., Zu P., Ang X.M., Wong W.C. (2012). Chitosan-coated polarization maintaining relative humidity measurement. IEEE J. Sel. Top. Quantum Electron..

[99] Mathew J., Semenova Y., Farrell G. A Miniature Optical Humidity Sensor.

[100] Mathew J., Semenova Y., Rajan G., Farrell G. (2010). Humidity sensor based on photonic crystal fibre interferometer. Electron. Lett..

[101] Liang H., Jin Y., Wang J., Dong X., Street X., Higher X., Zone E. (2012). Relative humidity sensor based on polarization maintainig fiber. Microw. Opt. Technol. Lett..

[102] Ivanov O., Nikitov S., Gulyaev Y. (2006). Cladding modes of optical fibers: properties and applications. Phys.-Uspekhi.

[103] Vasil'ev S.A., Dianov E.M., Medvedkov O.I., Protopopov V.N., Costantini D.M., Iocco A., Limberger H.G., Salathe R.P. (1999). Properties of the cladding modes of an optical fibre excited by refractive-index gratings. Quantum Electron..

[104] Alwis L., Sun T., Grattan K.T.V. (2013). Fibre optic long period grating-based humidity sensor probe using a Michelson interferometric arrangement. Sens. Actuators B Chem..

[105] Erdogan T. (1997). Cladding-mode resonances in short- and long-period fiber grating filters. J. Opt. Soc. Am. A.

[106] Fu M., Lin G., Liu W., Wu C. (2011). Fiber-optic humidity sensor based on an air-gap long period fiber grating. Opt. Rev..

[107] Konstantaki M., Pissadakis S., Pispas S., Madamopoulos N., Vainos N. (2006). Optical fiber long-period grating humidity sensor with poly(ethylene oxide)/cobalt chloride coating. Appl. Opt..

[108] Smietana M., Korwin-Pawlowski M.L., Bock W.J., Pickrell G.R., Szmidt J. (2008). Refractive index sensing of fiber optic long-period grating structures coated with a plasma deposited diamond-like carbon thin film. Meas. Sci. Technol..

[109] Wang L., Liu Y., Zhang M., Tu D., Mao X., Liao Y. (2007). A relative humidity sensor using a hydrogel-coated long period grating. Meas. Sci. Technol..

[110] Yu X., Childs P., Zhang M., Liao Y., Ju J., Jin W. (2009). Relative humidity sensor based on cascaded long-period gratings with hydrogel coatings and fourier demodulation. Photonics Technol. Lett. IEEE.

[111] Alwis L., Sun T., Grattan K.T.V. (2013). Analysis of polyimide-coated optical fiber long-period grating-based relative humidity sensor. IEEE Sens. J..

[112] Venugopalan T., Sun T., Grattan K. (2008). Long period grating-based humidity sensor for potential structural health monitoring. Sens. Actuators A Phys..

[113] Viegas D., Goicoechea J., Santos J.L., Araújo F.M., Ferreira L.A., Arregui F.J., Matias I.R. (2009). Sensitivity improvement of a humidity sensor based on silica nanospheres on a long-period fiber grating. Sensors.

[114] Mathew J., Semenova Y., Farrell G. (2013). Fiber optic hybrid device for simultaneous measurement of humidity and temperature. IEEE Sens. J..

[115] Marcuse D. (1991). Theory of Dielectric Optical Waveguides.

[116] Urrutia A., Goicoechea J., Rivero P.J., Matías I.R., Arregui F.J. (2013). Electrospun nanofiber mats for evanescent optical fiber sensors. Sens. Actuators B Chem..

[117] Aneesh R., Khijwania S.K. (2012). Titanium dioxide nanoparticle based optical fiber humidity sensor with linear response and enhanced sensitivity. Appl. Opt..

[118] Zhao Z., Duan Y. (2011). A low cost fiber-optic humidity sensor based on silica sol-gel film. Sens. Actuators B Chem..

[119] Akita S., Sasaki H., Watanabe K., Seki A. (2010). A humidity sensor based on a hetero-core optical fiber. Sens. Actuators B Chem..

[120] Xia L., Li L., Li W., Kou T., Liu D. (2013). Novel optical fiber humidity sensor based on a no-core fiber structure. Sens. Actuators A Phys..

[121] Zhao Y., Jin Y., Liang H. All-Fiber-Optic Sensor for Relative Humidity Measurement.

[122] Fuke M.V., Kanitkar P., Kulkarni M., Kale B.B., Aiyer R.C. (2010). Effect of particle size variation of Ag nanoparticles in Polyaniline composite on humidity sensing. Talanta.

[123] Liu Y., Zhang Y., Lei H., Song J., Chen H., Li B. (2012). Growth of well-arrayed ZnO nanorods on thinned silica fiber and application for humidity sensing. Opt. Express.

[124] Tao S., Winstead C., Jindal R., Singh J. (2004). Optical-fiber sensor using tailored porous sol-gel fiber core. IEEE Sens. J..

[125] Lee K., Wawro D., Priambodo P., Magnusson R. (2007). Agarose-gel based guided-mode resonance humidity sensor. IEEE Sens. J..

